# Supplementary material for: Hydrodynamics of metachronal paddling: effects of varying Reynolds number and phase lag
Source: R Soc Open Sci. 2019 Oct 16;6(10):191387. doi: 10.1098/rsos.191387 (PMC6837200; doi:10.1098/rsos.191387)
Supplement: Supplementary figures [file rsos191387supp1.pdf]

ELECTRONIC SUPPLEMENTARY MATERIAL

Supplementary figure captions

Figure S1. Time-varying hinge angles (β) for Re=50 tracked using ImageJ program. (A) 0% phase lag; (B) 16.7% phase lag; (C) 25% phase lag; (D) 33.3% phase lag. Time is represented as fraction of stroke period (T=0.667 s). For the tail-most paddle (P4 in inset of Figure 2A), PS is from 0 to 0.5 and RS is from 0.5 to 1.

Figure S2. Time-varying hinge angles (β) for Re=250 tracked using ImageJ program. (A) 0% phase lag; (B) 16.7% phase lag; (C) 25% phase lag; (D) 33.3% phase lag. Cycle duration, PS and RS portions are as described in caption of Figure S1.

Figure S3. Time-varying hinge angles (β) for Re=800 tracked using ImageJ program. (A) 0% phase lag; (B) 16.7% phase lag; (C) 25% phase lag; (D) 33.3% phase lag. Cycle duration, PS and RS portions are as described in caption of Figure S1.

Figure S4. Velocity vector fields overlaid on out-of-plane z-vorticity (ω_z) contours for metachronal paddling of four paddles, with 16.7% phase lag (A-H) and 33.3% phase lag (I-P), at Re=250. (A, I) 20% PS. (B, J) 40% PS. (C, K) 60% PS. (D, L) 80% PS. (E, M) 20% RS. (F, N) 40% RS. (G, O) 60% RS. (H, P) 80% RS. Red coloring represents counterclockwise vorticity, while blue represents clockwise vorticity. % PS and % RS are referenced with respect to right-most paddle (P4 in inset of Figure 2A) that is near the tail-end of the model.

Figure S5. Velocity vector fields overlaid on 2D divergence of velocity ($\nabla \cdot \vec{U} = \partial u/\partial \mathrm{x} + \partial v/\partial \mathrm{y}$) contours for synchronous paddling (0% phase lag) of four paddles at Re=250. (A) 20% PS. (B) 40% PS. (C) 60% PS. (D) 80% PS. (E) 20% RS. (F) 40% RS. (G) 60% RS. (H) 80% RS, referenced with respect to P4 (tail-most paddle, see inset of Figure 2A). 2D divergence is used to examine three-dimensionality of the flow field. Here, red coloring represents positive divergence (out-of-plane source), and blue coloring represents negative divergence (out-of-plane sink). Regions of positive divergence are generated behind the tail-most paddle at the end of recovery stroke during synchronous paddling (time period between H and A).

Figure S6. Velocity vector fields overlaid on 2D divergence of velocity ($\nabla \cdot \vec{U}$) contours for metachronal paddling of four paddles with 25% phase lag, at Re=250. Definitions of contour coloring, vector scaling, % PS, % RS are the same as in Figure S5. For metachronal paddling, regions of positive divergence are generated behind the tail-most paddle at the end of recovery

stroke (H), as well as between paddles when they are moving away from each other (e.g. between P2 and P3 in part E).

Figure S7. Velocity vector fields overlaid on 2D divergence of velocity ($\nabla \cdot \vec{U}$) contours for metachronal paddling of four paddles with 25% phase lag, at Re=50. Definitions of contour coloring, vector scaling, % PS, % RS are the same as in Figure S5. For Re=50, regions of positive divergence are generated in the same locations as at Re=250 (Figure S6), but are of smaller magnitude (less three-dimensionality of the flow).

Figure S8. Velocity vector fields overlaid on 2D divergence of velocity ($\nabla \cdot \vec{U}$) contours for metachronal paddling of four paddles with 25% phase lag, at Re=800. Definitions of contour coloring, vector scaling, % PS, % RS are the same as in Figure S5. For Re=800, regions of positive divergence are generated in the same locations as at Re=250 (Figure S6), but are of larger magnitude (more three-dimensionality of the flow).

Figure S9. Velocity vector fields overlaid on out-of-plane z-vorticity (ω_z) contours for synchronous paddling (0% phase lag) of four paddles at Re=50. (A) 20% PS. (B) 40% PS. (C) 60% PS. (D) 80% PS. (E) 20% RS. (F) 40% RS. (G) 60% RS. (H) 80% RS. Red coloring represents counterclockwise vorticity, while blue represents clockwise vorticity. % PS and % RS are referenced with respect to P4 in inset of Figure 2A.

Figure S10. Velocity vector fields overlaid on out-of-plane z-vorticity (ω_z) contours for metachronal paddling of four paddles at 16.7% phase lag, at Re=50. Definitions of contour coloring, % PS, % RS are the same as in Figure S9.

Figure S11. Velocity vector fields overlaid on out-of-plane z-vorticity (ω_z) contours for metachronal paddling of four paddles at 33.3% phase lag, at Re=50. Definitions of contour coloring, % PS, % RS are the same as in Figure S9.

Figure S12. Velocity vector fields overlaid on out-of-plane z-vorticity (ω_z) contours for synchronous paddling (0% phase lag) of four paddles, at Re=800. Definitions of contour coloring, % PS, % RS are the same as in Figure S9.

Figure S13. Velocity vector fields overlaid on out-of-plane z-vorticity (ω_z) contours for metachronal paddling of four paddles at 16.7% phase lag, at Re=800. Definitions of contour coloring, % PS, % RS are the same as in Figure S9.

Figure S14. Velocity vector fields overlaid on out-of-plane z-vorticity (ω_z) contours for metachronal paddling of four paddles at 33.3% phase lag, at Re=800. Definitions of contour coloring, % PS, % RS are the same as in Figure S9.

Figure S1

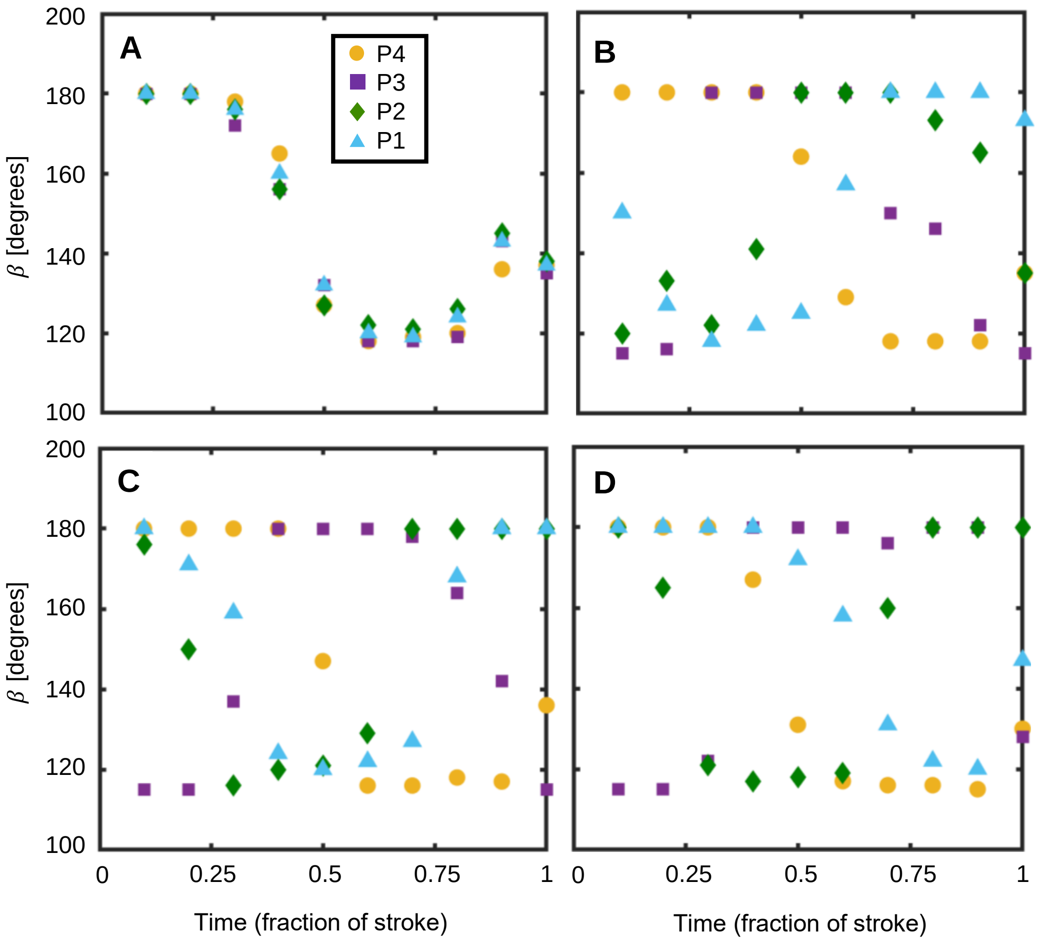
A
B
C
D
P4
P3
P2
P1
200
180
160
140
120
100
β [degrees]
0
0.25
0.5
0.75
1
Time (fraction of stroke)

Figure S2

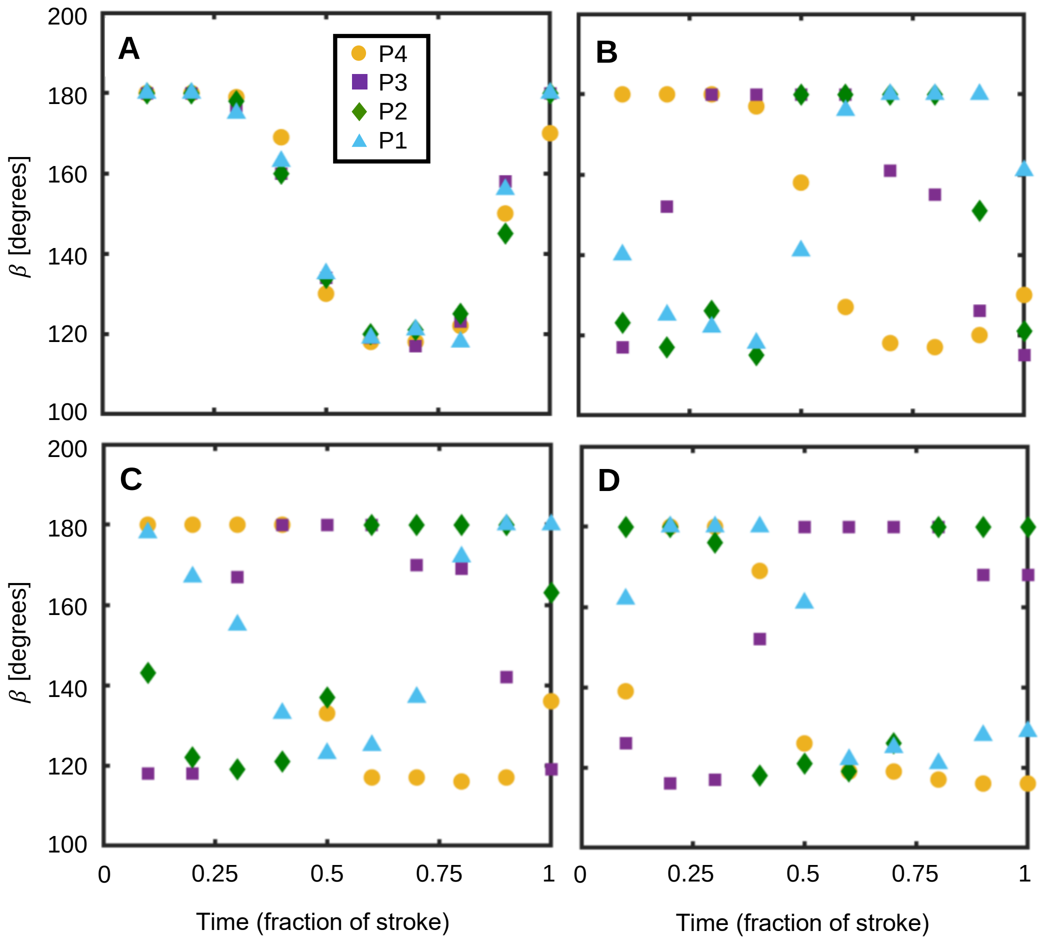

A
B
C
D
P4
P3
P2
P1
β [degrees]
200
180
160
140
120
100
0
0.25
0.5
0.75
1
Time (fraction of stroke)

Figure S3

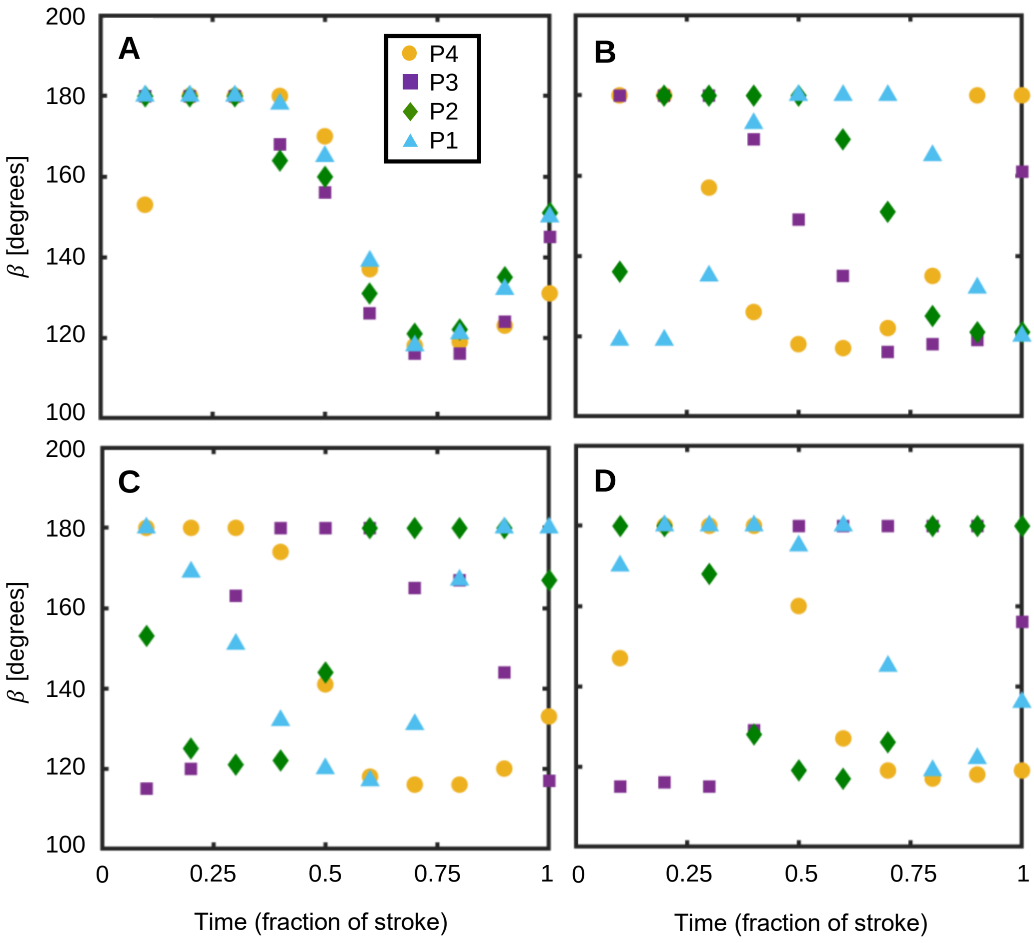
A
B
C
D
P4
P3
P2
P1
200
180
160
140
120
100
β [degrees]
0
0.25
0.5
0.75
1
Time (fraction of stroke)

Figure S4

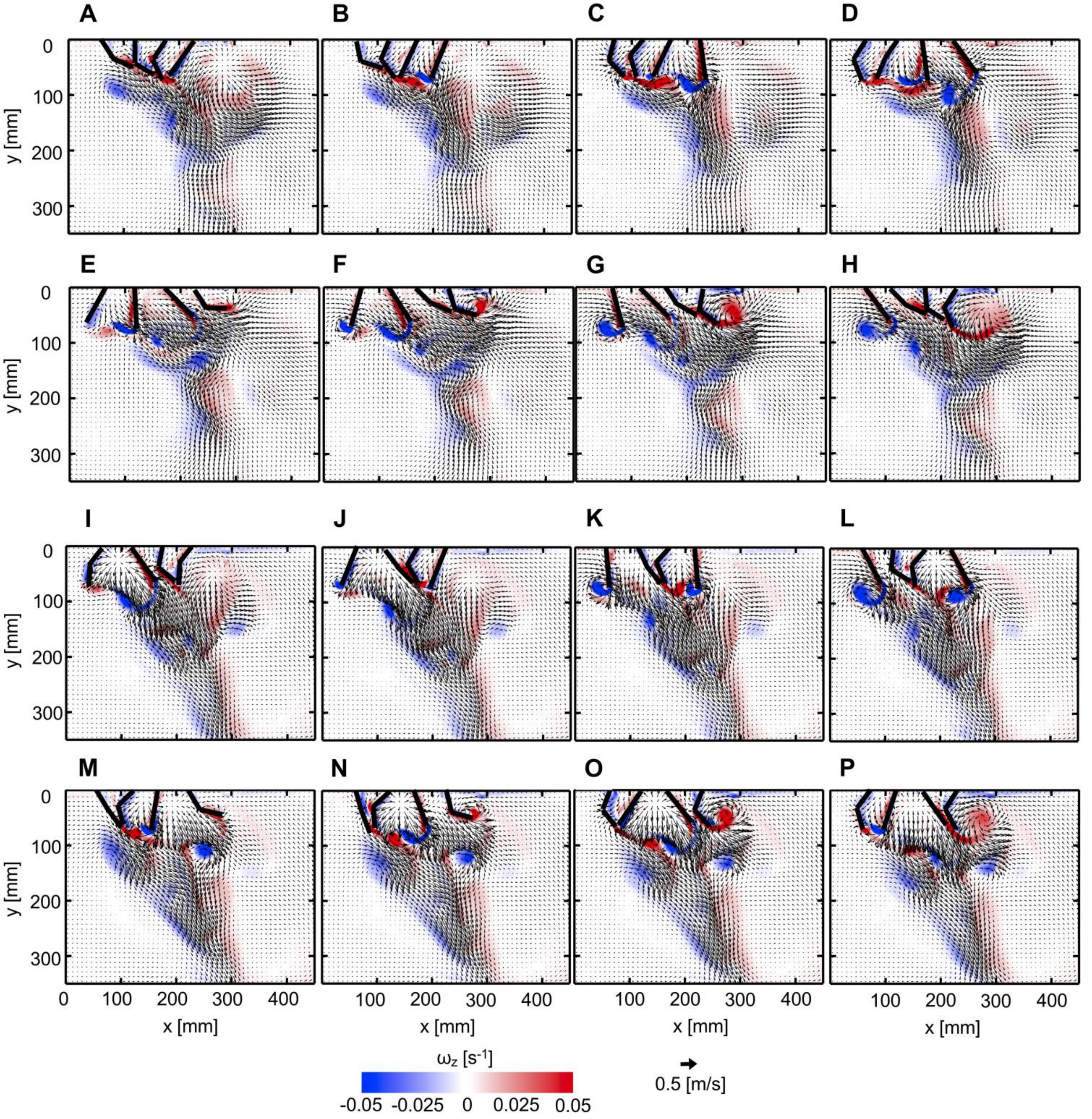

A
B
C
D
E
F
G
H
I
J
K
L
M
N
O
P
y [mm]
x [mm]
0
100
200
300
400
ωz [s-1]
-0.05
-0.025
0
0.025
0.05
0.5 [m/s]

Figure S5

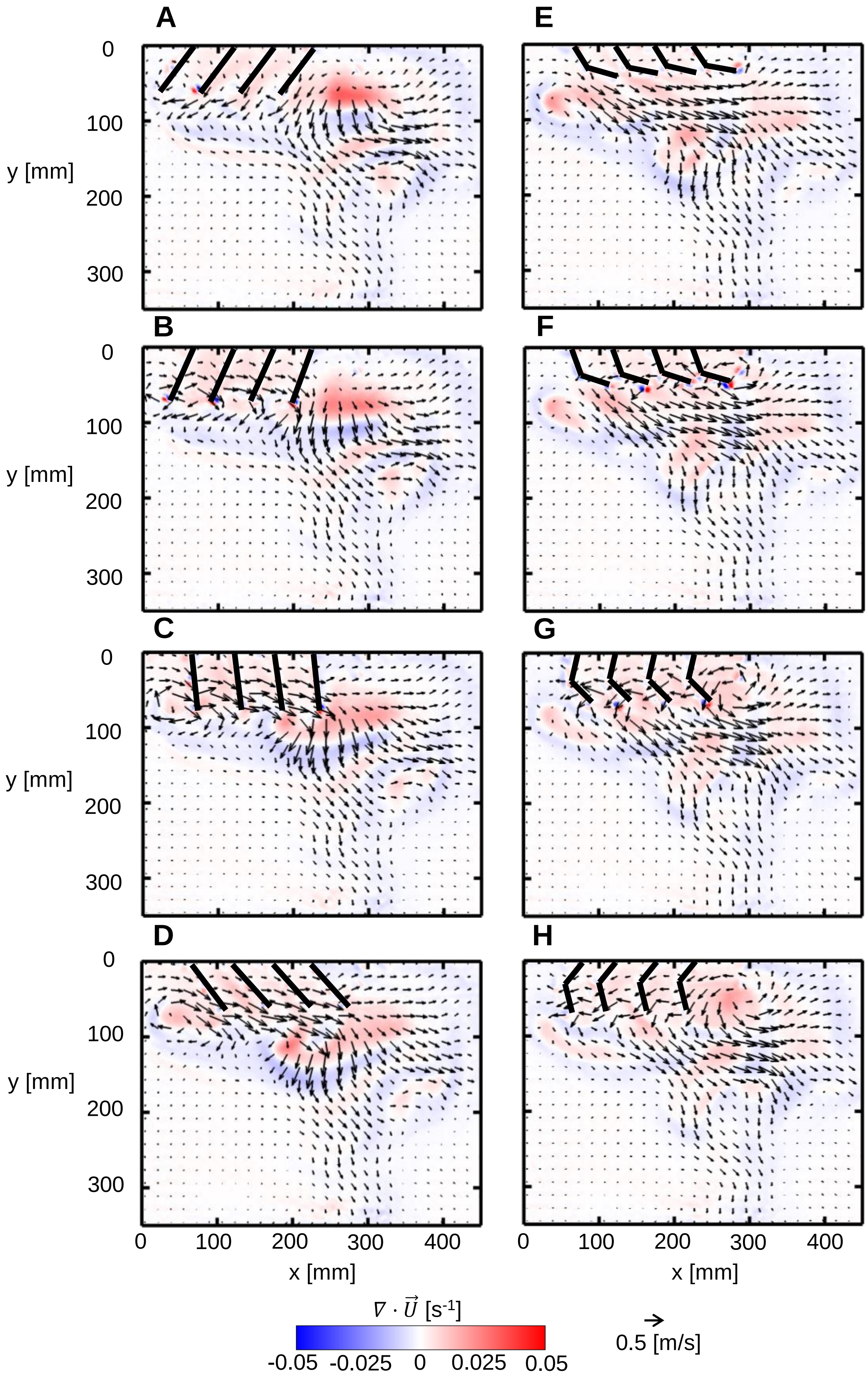

A
B
C
D
E
F
G
H
0
100
200
300
y [mm]
0
100
200
300
400
x [mm]
$\nabla \cdot \vec{U}$ [s^{-1}]
-0.05
-0.025
0
0.025
0.05
0.5 [m/s]

Figure S6

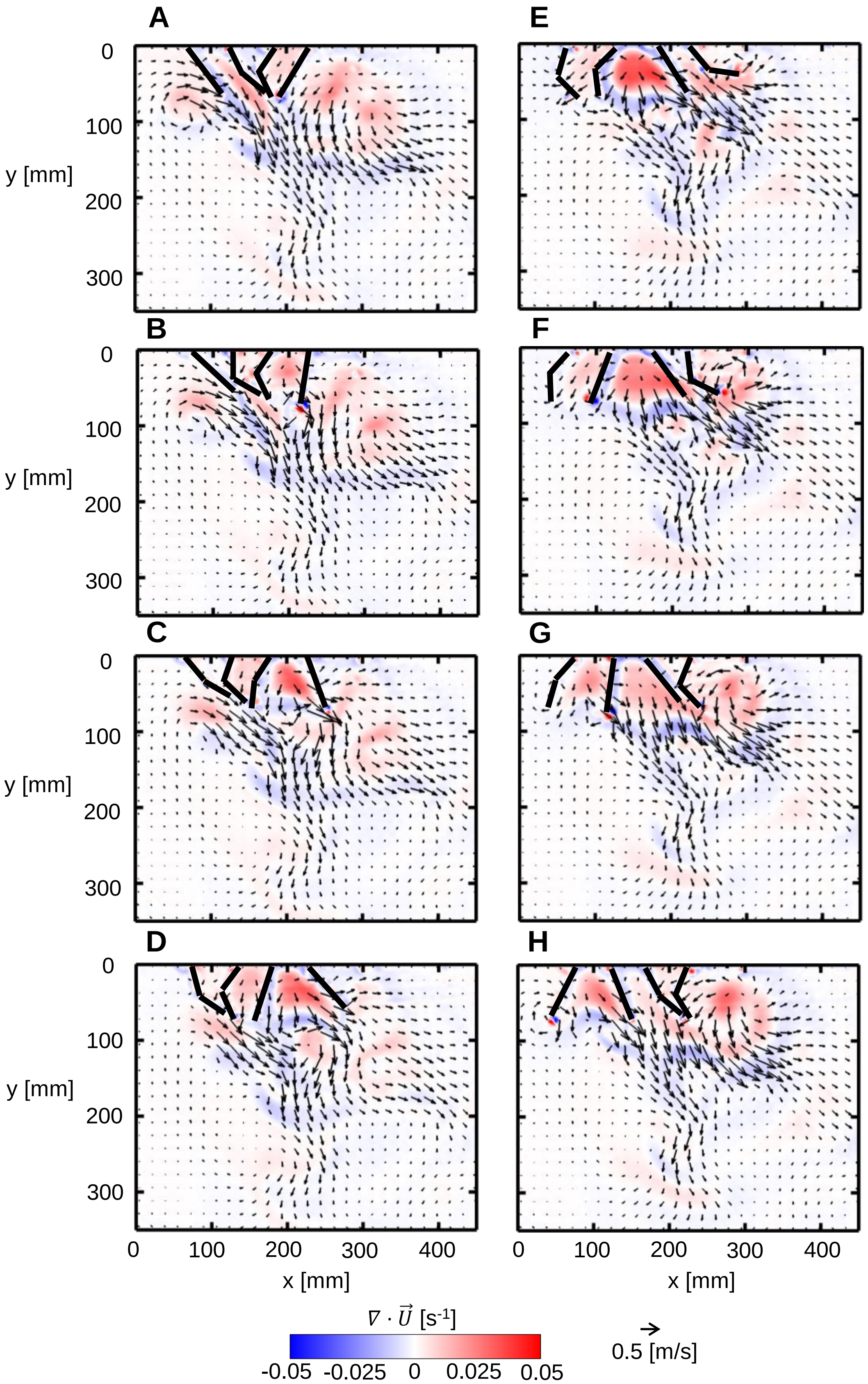

A
B
C
D
E
F
G
H
y [mm]
x [mm]
0
100
200
300
400
$\nabla \cdot \vec{U}$ [s^{-1}]
-0.05
-0.025
0
0.025
0.05
0.5 [m/s]

Figure S7

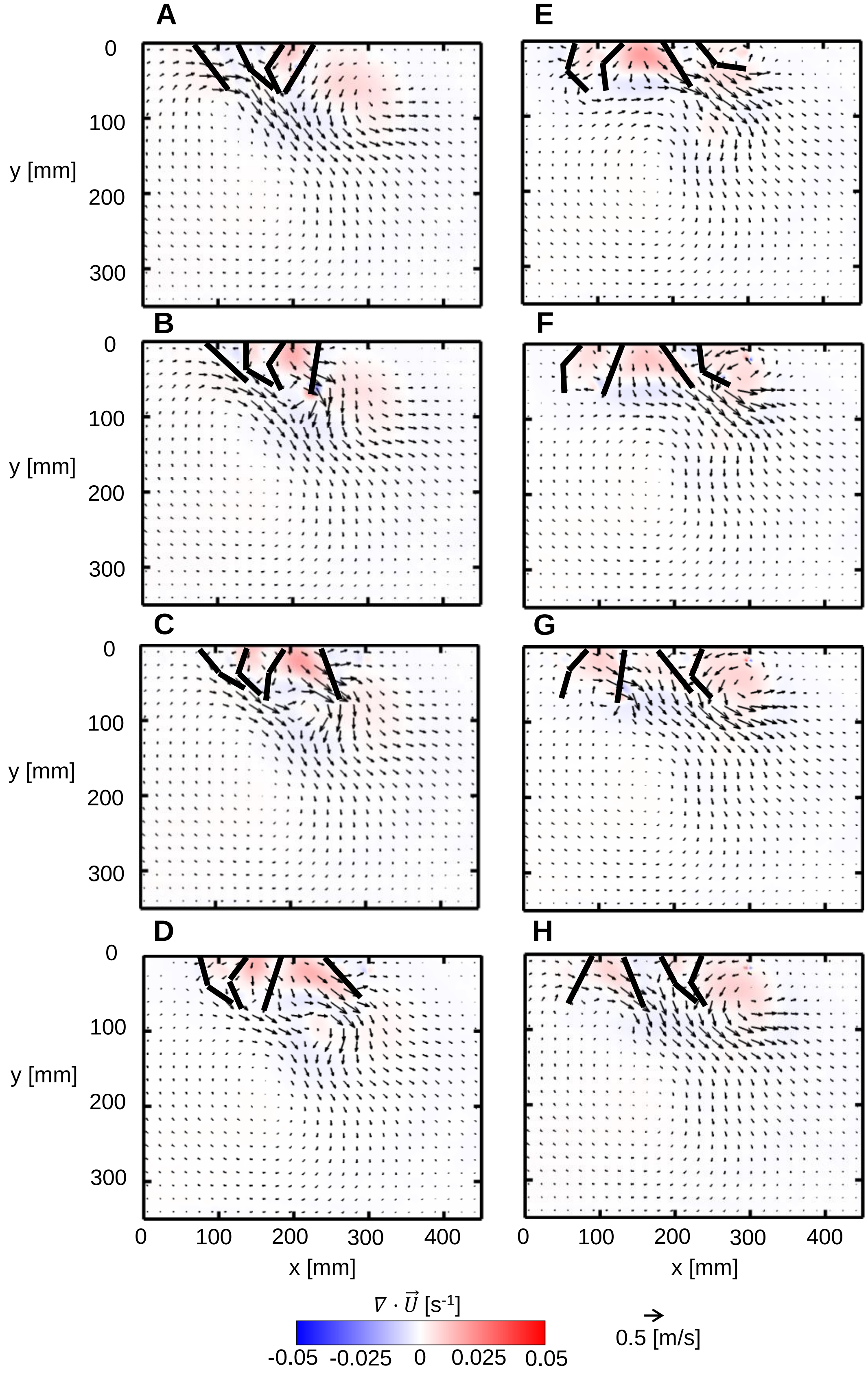
A
B
C
D
E
F
G
H
y [mm]
x [mm]
0
100
200
300
400
$\nabla \cdot \vec{U}$ [s-1]
-0.05
-0.025
0
0.025
0.05
0.5 [m/s]

Figure S8

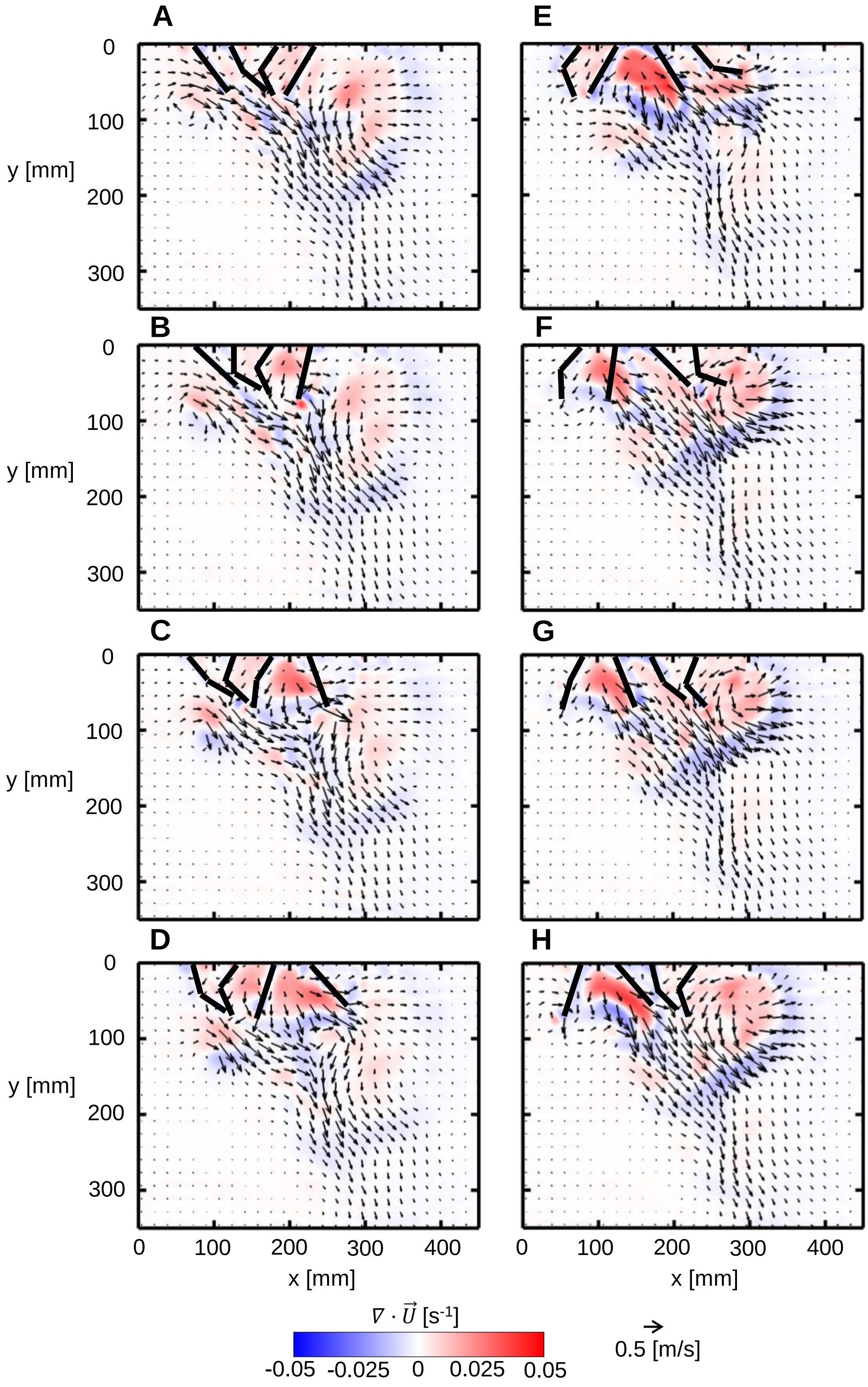
A
B
C
D
E
F
G
H
y [mm]
0
100
200
300
x [mm]
400
$\nabla \cdot \vec{U}$ [s-1]
-0.05
-0.025
0.025
0.05
0.5 [m/s]

Figure S9

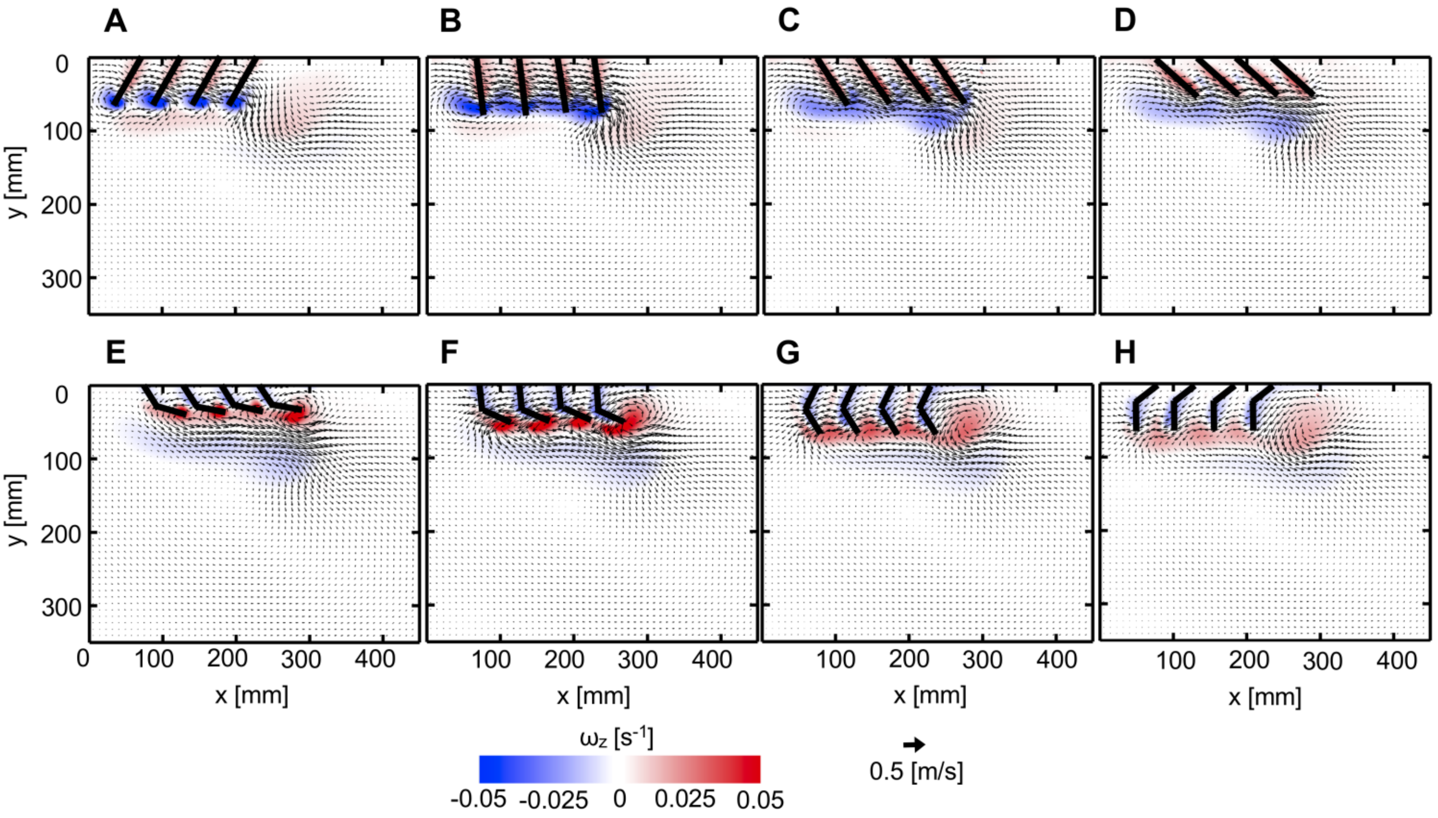
A
B
C
D
E
F
G
H
y [mm]
x [mm]
0
100
200
300
400
ωz [s-1]
-0.05
-0.025
0
0.025
0.05
0.5 [m/s]

Figure S10

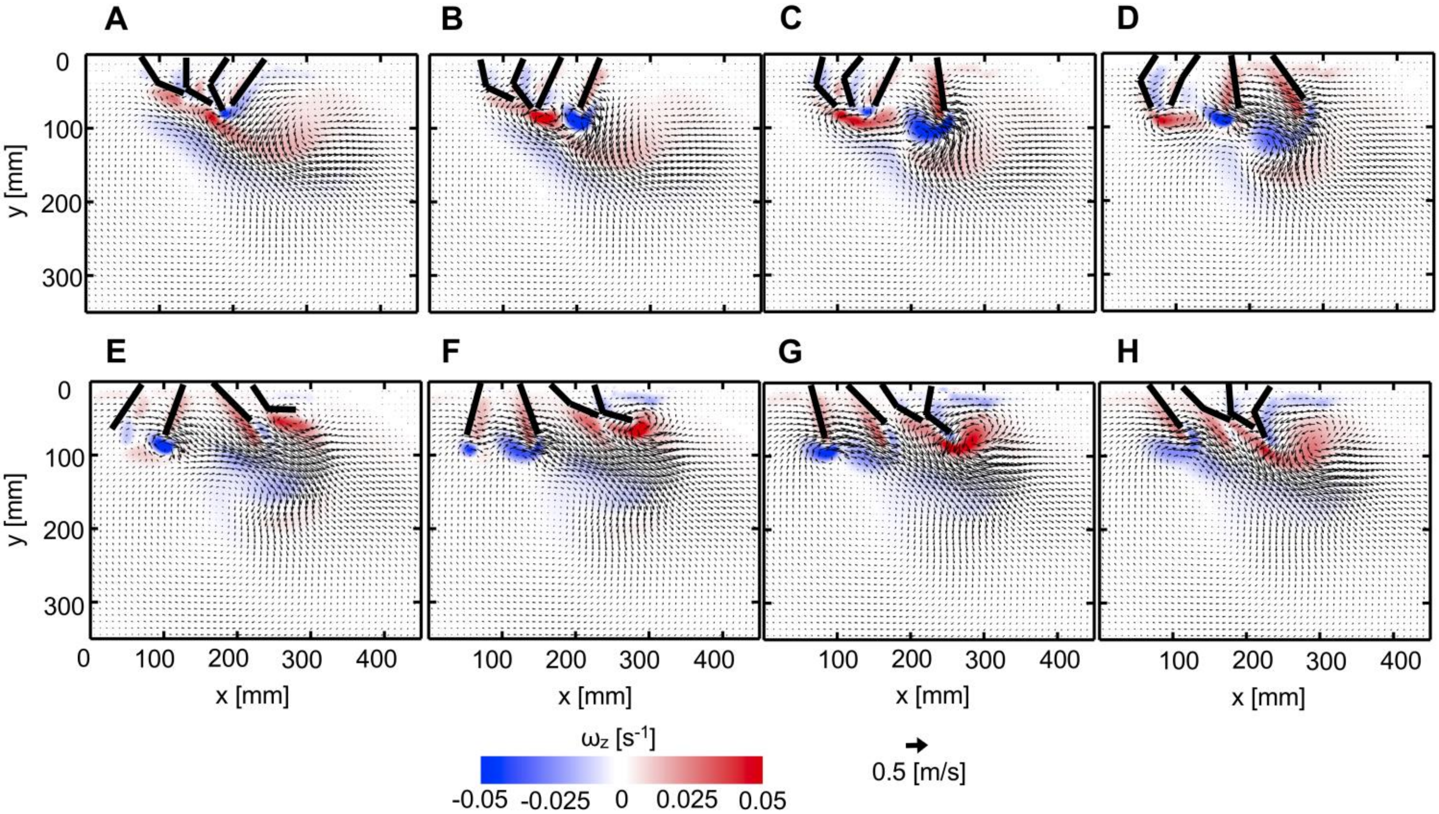

A
B
C
D
E
F
G
H
0
100
200
300
y [mm]
0
100
200
300
400
x [mm]
ωz [s-1]
-0.05
-0.025
0
0.025
0.05
0.5 [m/s]

Figure S11

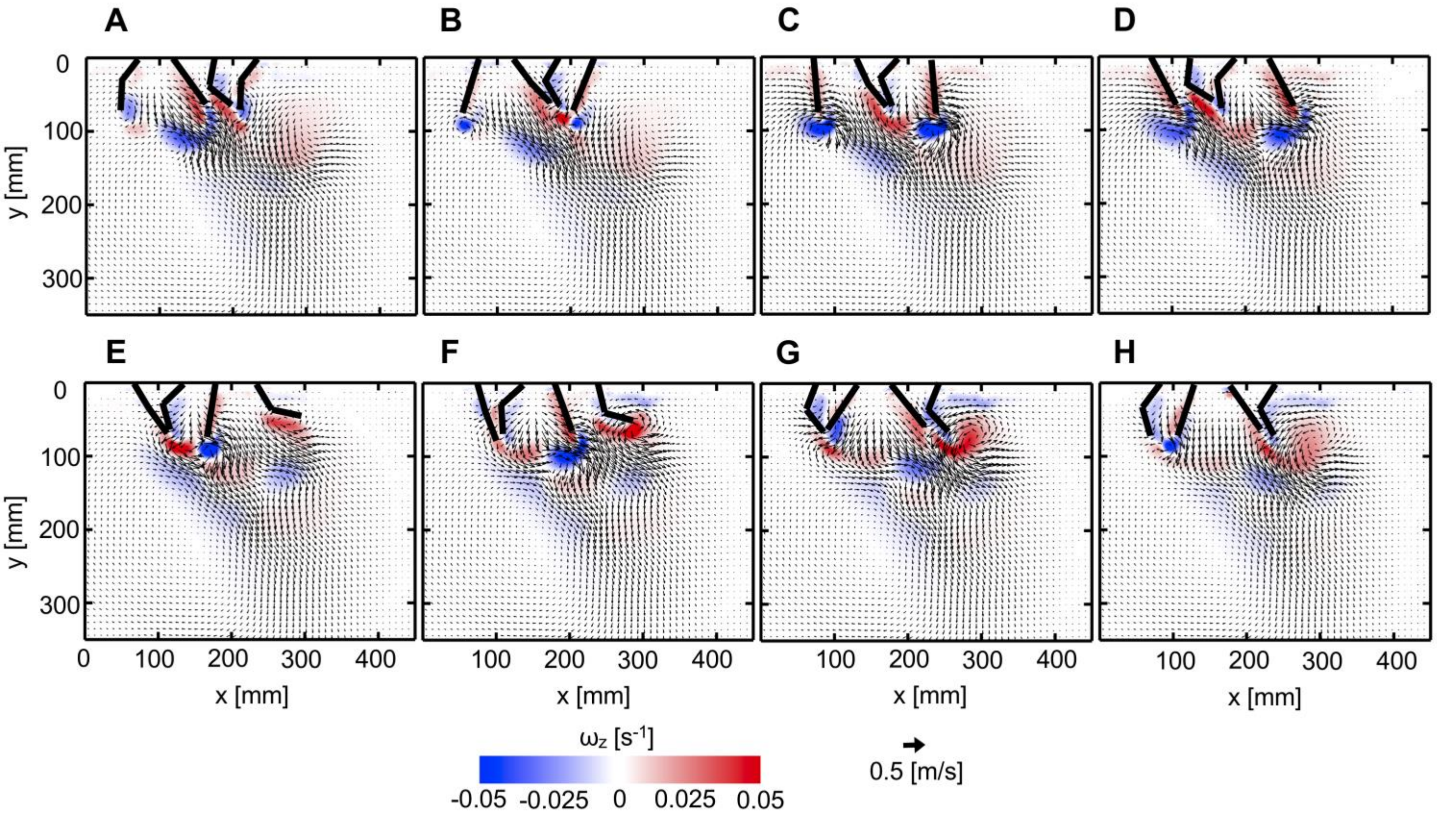

A
B
C
D
E
F
G
H
y [mm]
x [mm]
0
100
200
300
400
ωz [s-1]
-0.05 -0.025 0 0.025 0.05
0.5 [m/s]

Figure S12

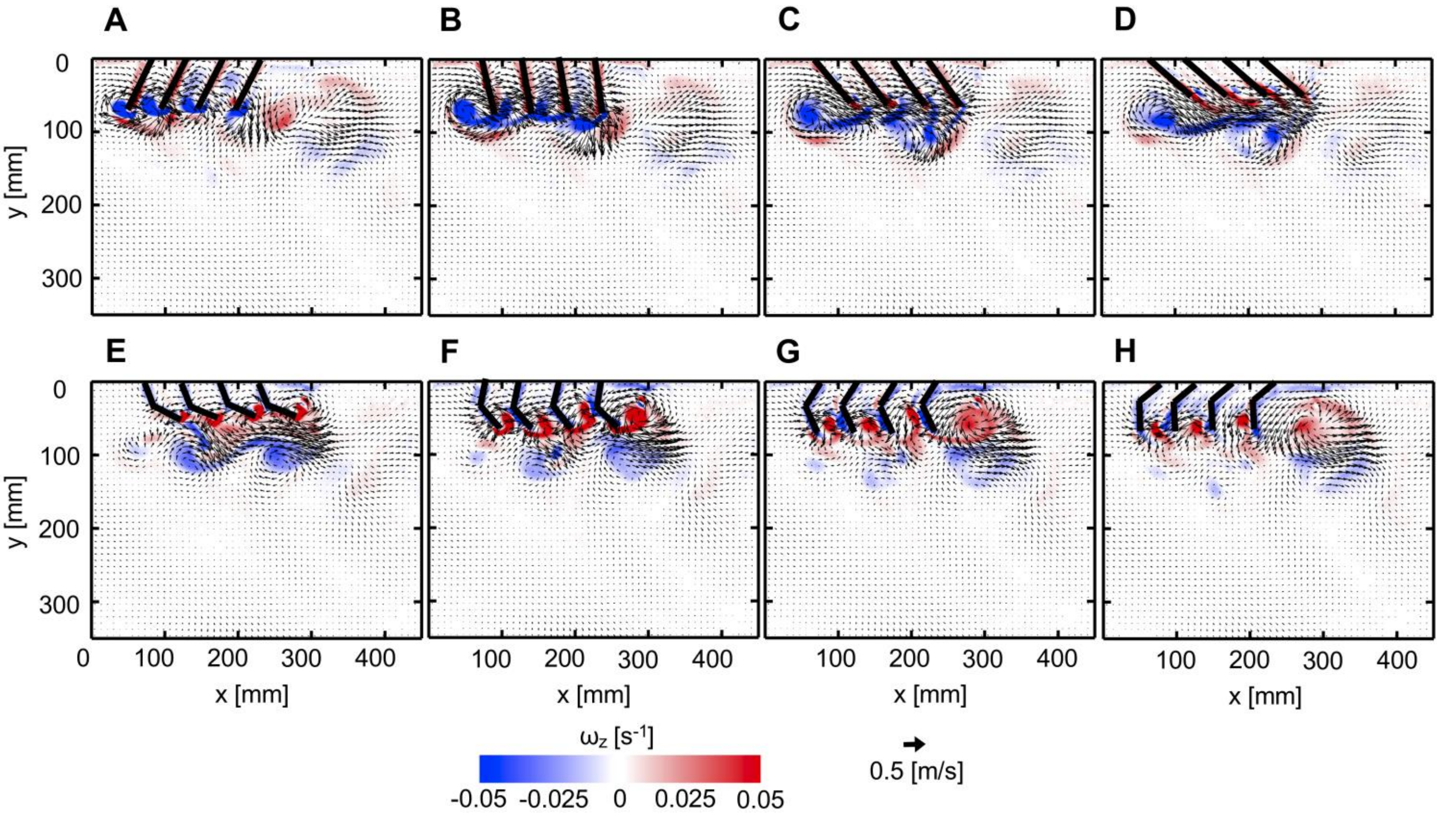
A
B
C
D
E
F
G
H
y [mm]
x [mm]
0
100
200
300
400
ω_z [s^{-1}]
-0.05
-0.025
0
0.025
0.05
0.5 [m/s]

Figure S13

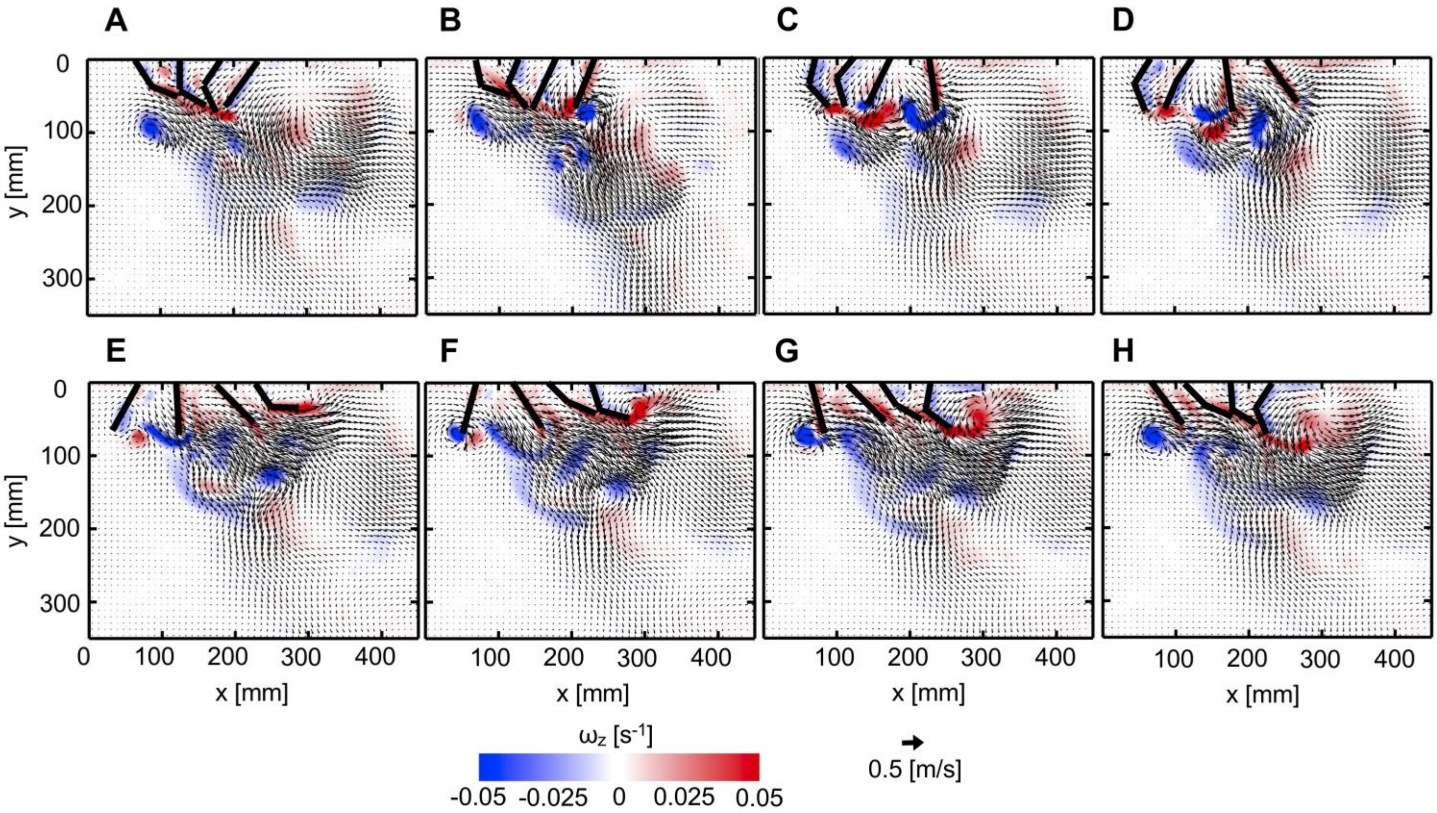

A
B
C
D
E
F
G
H
y [mm]
0
100
200
300
x [mm]
0
100
200
300
400
ωz [s-1]
-0.05
-0.025
0
0.025
0.05
0.5 [m/s]

Figure S14

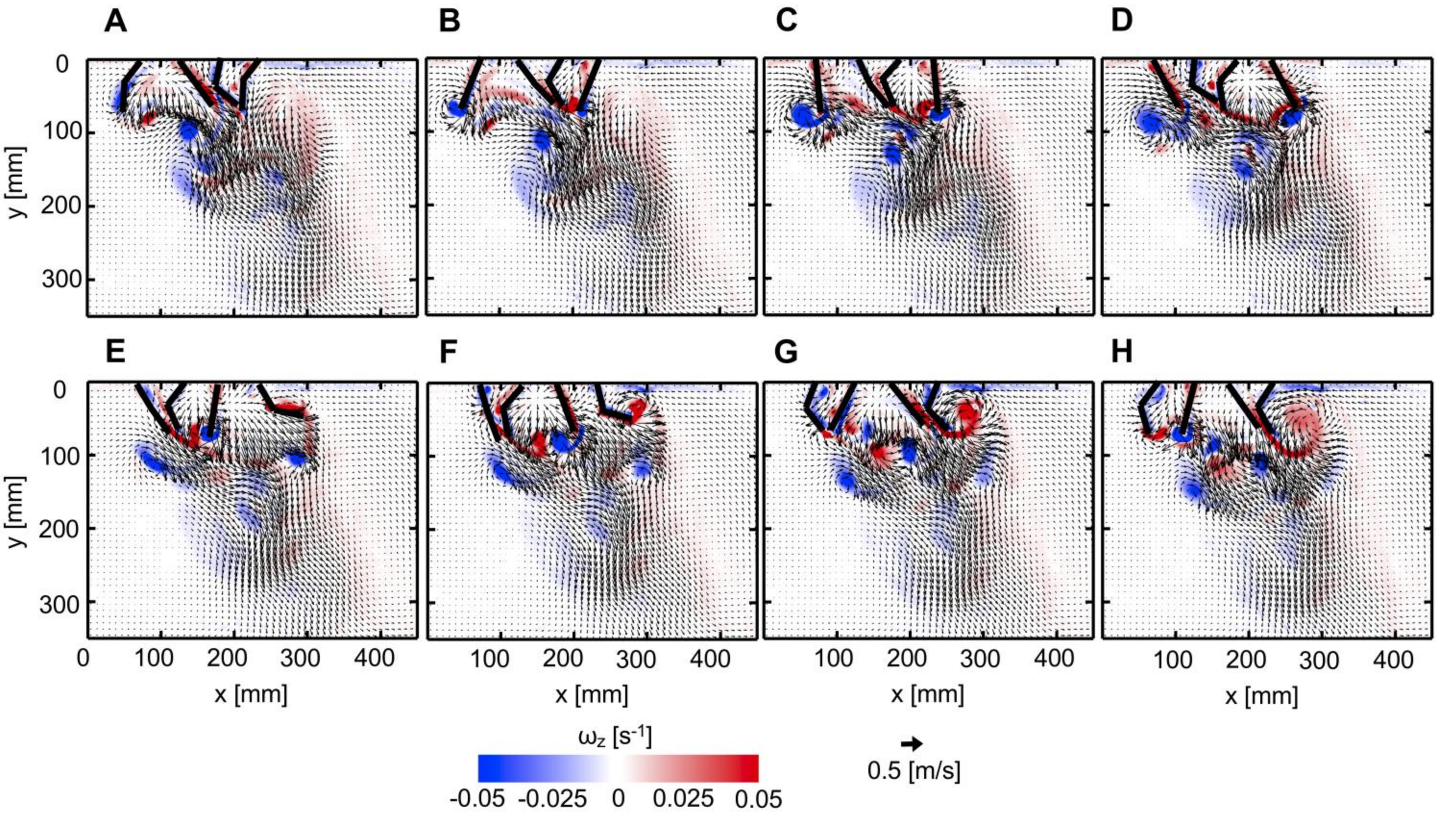

A
B
C
D
E
F
G
H
y [mm]
x [mm]
0
100
200
300
400
ω_z [s^{-1}]
-0.05
-0.025
0
0.025
0.05
0.5 [m/s]
